# Case report and literature review: tislelizumab combined with lenvatinib and polymeric micellar paclitaxel for thymic squamous cell carcinoma

**DOI:** 10.3389/fimmu.2025.1677723

**Published:** 2025-09-18

**Authors:** Jingwen Chen, Yihan Wang, Minyue Shou, Jieyi Qian, Ling Ma, Yongqian Shu

**Affiliations:** ^1^ Department of Oncology, The First Affiliated Hospital with Nanjing Medical University, Nanjing Medical University, Nanjing, Jiangsu, China; ^2^ Collaborative Innovation Center for Cancer Personalized Medicine, Nanjing Medical University, Nanjing, China; ^3^ Department of Oncology, the Affiliated Wuxi People’s Hospital of Nanjing Medical University, Wuxi, China

**Keywords:** advanced thymic squamous cell carcinoma, immunotherapy, irAEs, case report, literature review

## Abstract

Thymic squamous cell carcinoma (TSCC) is a rare malignancy with an annual incidence of 0.15–0.32 per 100,000 population and exhibits aggressive behavior including early metastasis. Anti-PD-1 immunotherapy combined with chemotherapy has emerged as a potential strategy for TSCC. We report a 60-year-old male with a 1-month history of persistent dry cough and generalized fatigue. Diagnostic evaluation revealed advanced TSCC (cT3N2M1b), with bilateral lung, right pleural, left adrenal, mediastinal and right hilar lymph nodes metastases. Significantly elevated CA125 and CYFRA211 levels were observed. Initial first-line carboplatin plus polymeric micellar paclitaxel (PM-PTX) stabilized the disease. Subsequent adjustments to the immunotherapy regimen led to a two-phase approach combining tislelizumab, lenvatinib, PM-PTX, with or without carboplatin. This strategy achieved sustained tumor burden reduction with improved quality of life and nutritional status throughout treatment, without significant immune-related adverse events (irAEs). Additionally, we discuss the mechanisms of combination therapy, immunotherapy toxicities in thymic malignancies, and propose personalized combination strategies to guide clinicians in selecting treatment options.

## Introduction

Thymic carcinoma is a rare anterior mediastinal malignancy with an annual incidence of 0.07 to 0.38/100,000 and a 55% 5-year survival rate (1, 2). Thymic squamous cell carcinoma (TSCC), its most common histological subtype, arises from thymic epithelial cells with aggressive behavior (1). TSCC typically expresses CD5, CK5, p40, and p63 by immunohistochemistry (IHC) (3), and frequently invades adjacent structures such as pericardium, lungs, phrenic nerve, and major blood vessels, or metastasizes to regional lymph nodes and extrathoracic sites (2). While localized TSCC is managed surgically alone or in combination with radiotherapy and chemotherapy, approximately 68% present with metastasis at the time of diagnosis. Platinum-based chemotherapy remains standard for unresectable disease (3, 4).

Immune checkpoint inhibitors targeting PD-1/PD-L1 have shown promise in thymic carcinoma. Giaccone et al. reported pembrolizumab’s efficacy as second-line therapy, albeit with higher incidence of severe immune-related adverse events (irAEs, 15%) compared to other tumor types (5). Lenvatinib, a multi-target inhibitor of VEGFR, FGFR, RET, c-Kit, and other kinases, also demonstrates a positive effect on advanced cases (6). Optimal immune-combination strategies for advanced TSCC require further exploration.

Here, we describe an advanced metastatic TSCC patient successfully treated with tislelizumab (anti-PD-1), lenvatinib, and polymeric micellar paclitaxel (PM-PTX), achieving tumor reduction while preserving quality of life through regimen adjustment. We also review the mechanisms underlying the enhanced anti-tumor efficacy of combination therapy, analyze patient selection criteria suitable for immunotherapy, and propose personalized combination strategies.

## Case presentation

**Figure 1:**
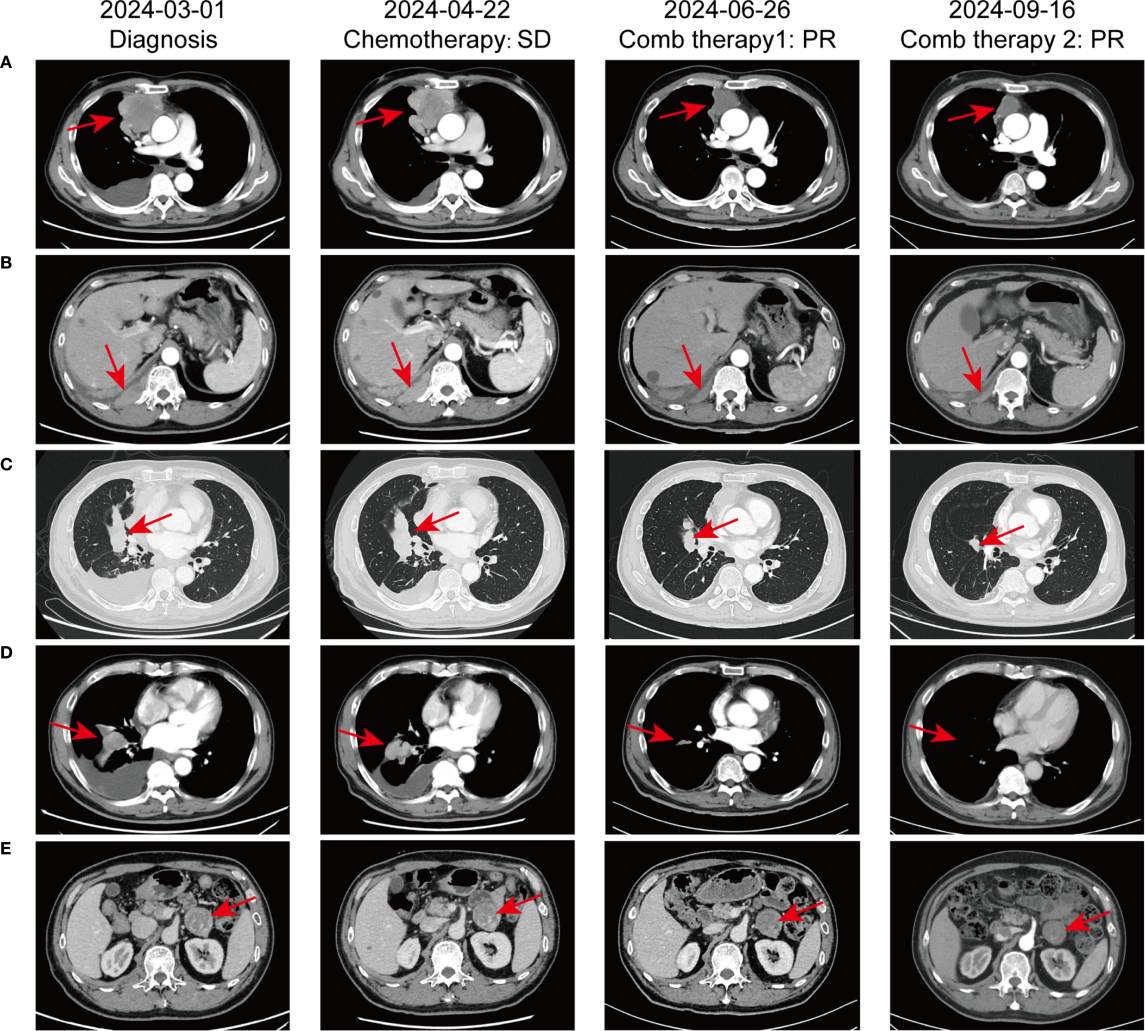
Comparative chest CT scans illustrating the progression of pulmonary lesions throughout the patient’s treatment course. **(A)** Irregular mass-like soft tissue shadow in the anterior superior mediastinum; **(B)** Mass-like soft tissue shadow in the right pleura; **(C)** Mass in the right middle lobe of the lung; **(D)** Multiple lymph node metastases in the mediastinum and right hilar lymph nodes; **(E)** Metastasis to the left adrenal gland.

**Figure 2:**
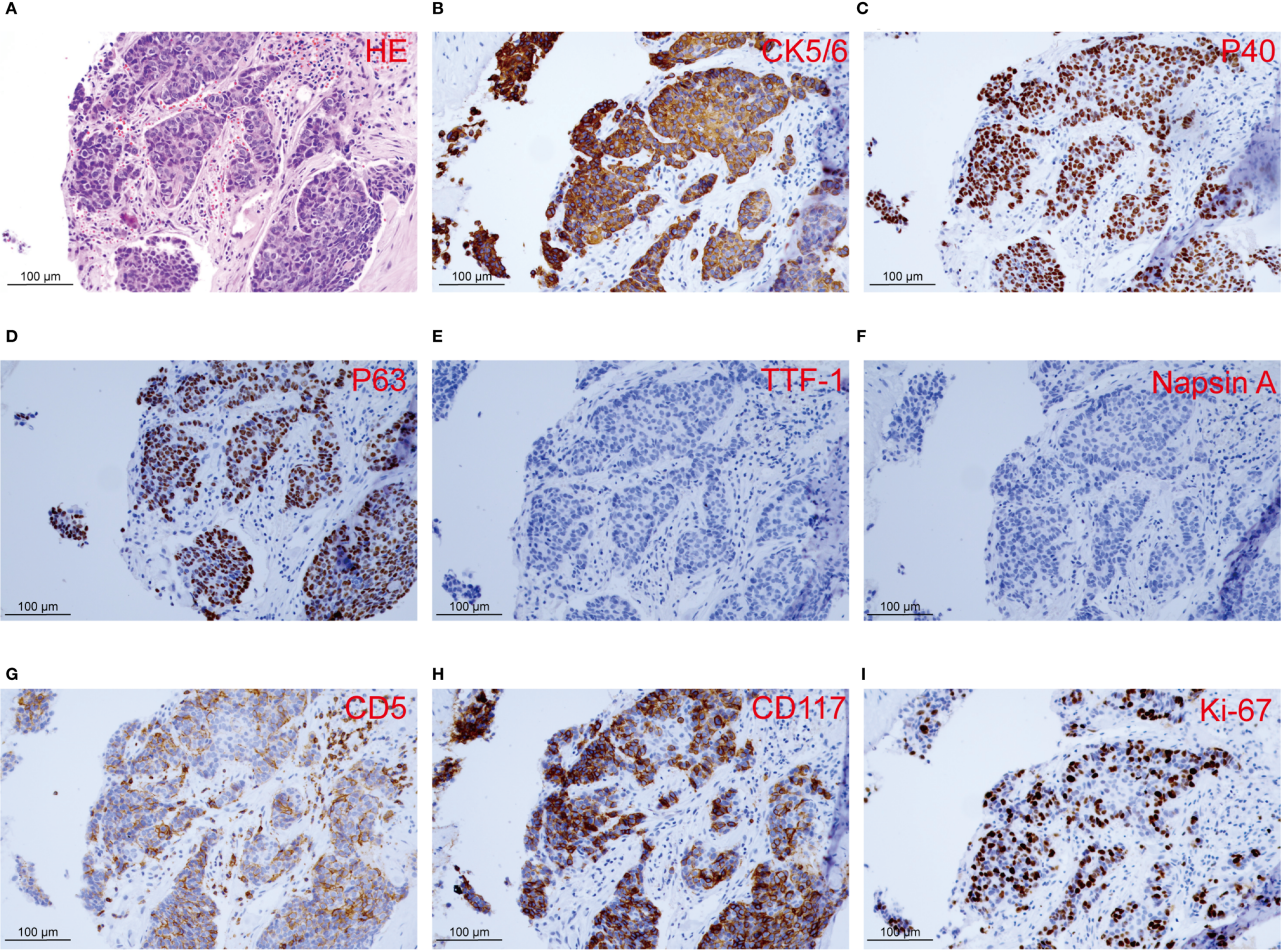
Histopathology and immunohistochemistry of the right lung lesion observed in the patient. **(A)** Histological section with HE staining reveals invasive carcinoma. **(B-I)** IHC staining of CK5/6, P40, P63, TTF-1, Napsin A, CD5, CD117, and Ki-67.

**Figure 3:**
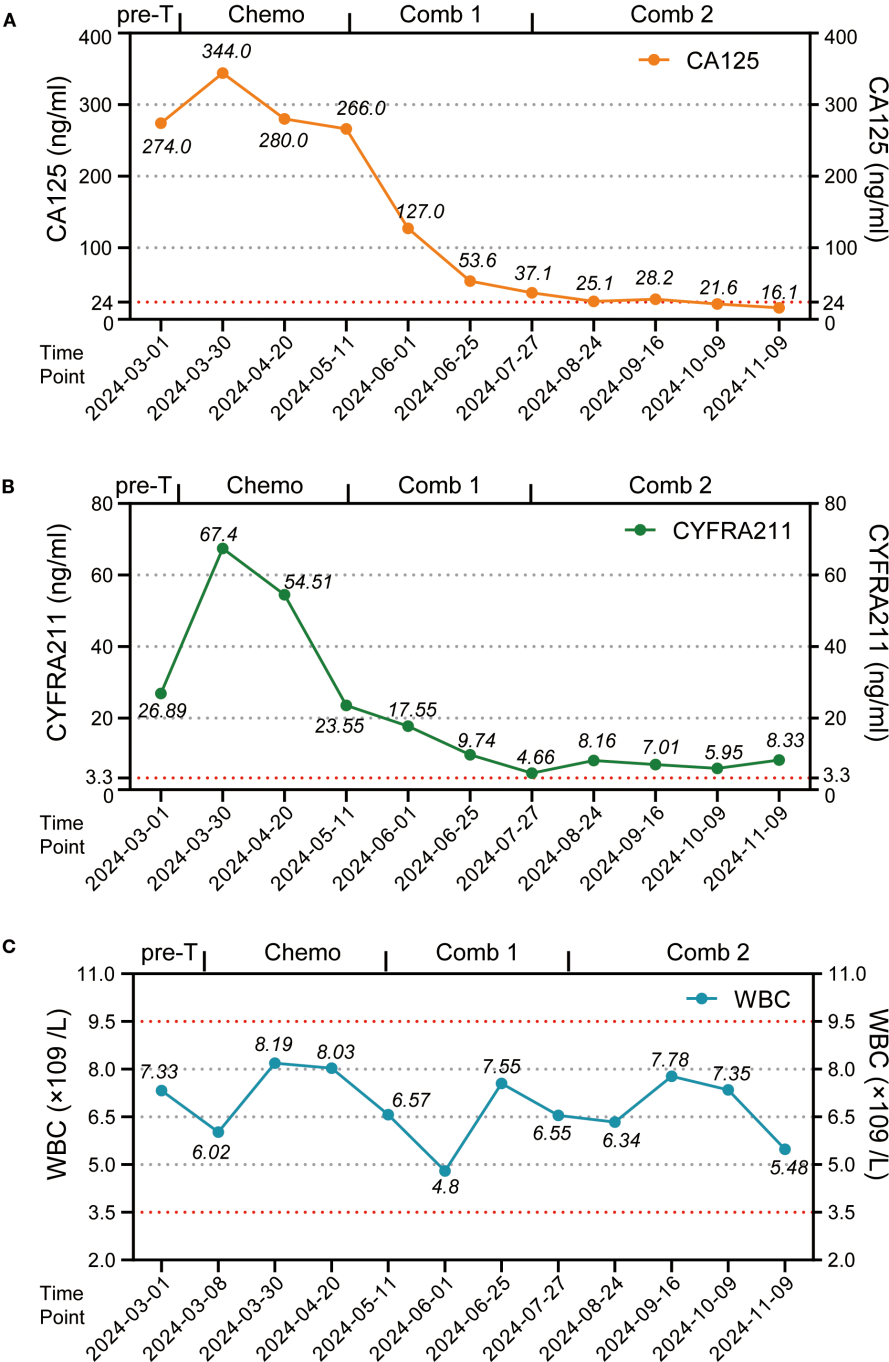
The variations in tumor markers and leukocyte counts during patient treatment. **(A)** Point-line plot of tumor marker CA125 levels. **(B)** Point-line plot of tumor marker CYFRA 21–1 levels. **(C)** Point-line plot of WBC counts. Pre-T, pre-treatment; Chemo, chemotherapy; Comb 1, combination therapy stage 1; Comb 2, combination therapy stage 2; CA125, Cancer Antigen 125; CYFRA211, Cytokeratin 19 fragment antigen 21-1.

In February 2024, a 60-year-old male patient presented with a 1-month history of a dry cough and fatigue without an apparent cause, which prompted a visit to a local hospital. A chest computed tomography (CT) scan revealed a right lung mass. For further evaluation and treatment, the patient was admitted to our oncology department on March 1, 2024. Enhanced chest and whole-abdomen CT scan showed a space-occupying lesion in the right middle lobe (RML) of the lung, multiple solid nodules in both lungs, multiple soft tissue density shadows in the right pleura, an inhomogeneous enhancing lesion in the left adrenal gland, a mass-like soft tissue density shadow in the anterior mediastinum, and multiple enlarged lymph nodes in the mediastinum and right hilum (**Figure 1**). On March 5, 2024, CT-guided right lung mass biopsy indicated invasive carcinoma. IHC showed CK5/6(+), P40(+), P63(+), TTF-1(-), Napsin A(-), CD5(+), and CD117(+), with a Ki-67 ~35% (**Figure 2**). Multidisciplinary diagnosis confirmed advanced thymic squamous cell carcinoma, with metastasis to lung, right pleura, left adrenal gland, and mediastinal and right hilar lymph nodes (cT3N2M1b, Stage IVB). Biomarker and NGS analysis revealed PD-L1 negativity (TPS<1%, details in **Supplementary Table 1**). Tumor markers abnormally elevated: CA125–274 ng/mL (ref: 0-24.00), CYFRA211 26.89 ng/mL (ref: 0-3.3) (**Figure 3**).

For unresectable disease, the patient underwent three cycles of first-line chemotherapy from March 7 to April 20, 2024: PM-PTX 360 mg D1 q3w + carboplatin 450 mg D1 q3w. A follow-up chest CT on April 22 showed largely unchanged lesions after treatment, resulting in an efficacy evaluation of stable disease (SD) (**Figure 1**). CA125 and CYFRA211 remained elevated (**Figure 3**). The patient experienced tolerable chemotherapy side effects, including decreased appetite and slight weight loss.

To reduce tumor burden, we evaluated the feasibility of immunotherapy and oral anti-angiogenic agents. Based on the REMORA study, lenvatinib has been shown to provide survival benefits for thymic carcinoma patients who have undergone first-line platinum-based chemotherapy (7). Consequently, from May 11 to June 25, 2024, the patient received three cycles of PM-PTX 360 mg D1 q3w + carboplatin 450 mg D1 q3w + tislelizumab 200 mg D1 q3w + lenvatinib 8 mg qd. CT on June 26, 2024, showed reduction of both primary and metastatic lesions. The efficacy evaluation indicated a partial response (PR), confirming the initial effectiveness of the added immunotherapy. CA125 significantly decreased to 53.6 ng/mL, CYFRA211 to 9.74 ng/mL; WBC level remained within the normal range (**Figure 3**). Throughout the treatment period, the patient maintained a positive mental state, experienced reduced toxic side effects from the medications, and no adverse reactions to the immunotherapy were observed.

**Figure 4:**
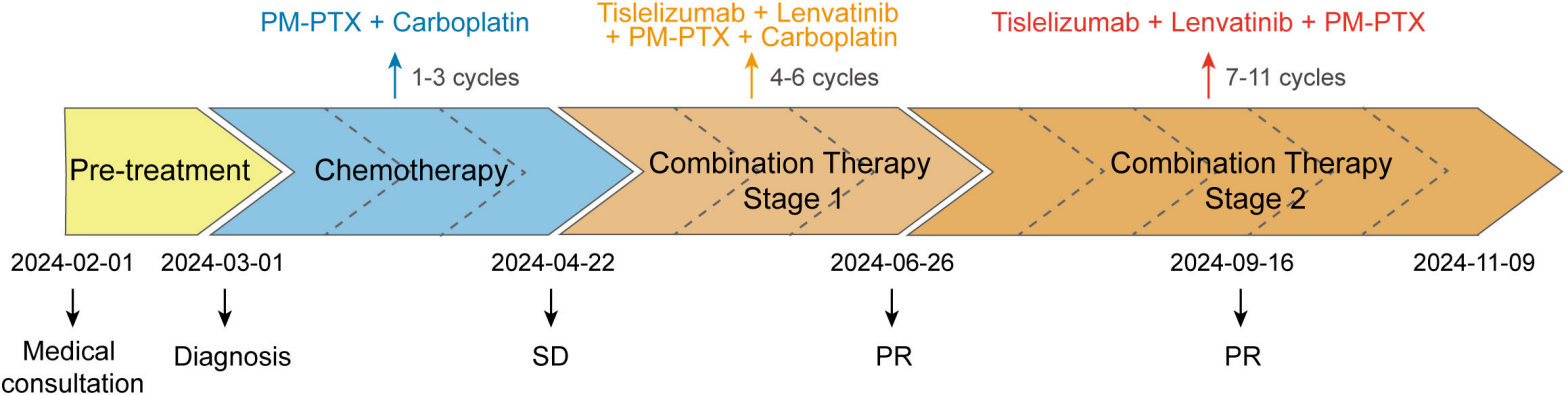
Medical intervention schedule. SD, stable disease; PR, partial response.

Given the cumulative toxicity resulting from the long-term use of chemotherapeutic drugs, carboplatin was discontinued. From July 27 to November 9, 2024, the patient underwent five cycles of PM-PTX 360mg D1 q3w + tislelizumab 200mg D1 q3w + lenvatinib 8mg qd. After three cycles of combination therapy, CT on September 16 revealed further shrinkage of the primary and metastatic lesions compared to June 25, with minor chronic inflammation observed in the right lung and the lower lobe of the left lung. The efficacy was evaluated as PR, confirming that the combination therapy has effectively controlled the patient’s condition. CA125 and CYFRA211 neared normal, and WBC remains within the normal range (**Figure 3**). During the five cycles of combined treatment, the patient demonstrated an improved mental state, further alleviation of clinical symptoms, and steady weight gain. The toxic side effects of anti-tumor treatments, particularly immunotherapy, can significantly impact the patient’s quality of life and adherence to treatment (8). However, over total eight immunotherapy cycles administered in two phases, the patient experienced no grade ≥3 irAEs. Only grade 1–2 toxicities were observed, including forearm erythema, abdominal discomfort, and mildly elevated transaminases (**Supplementary Table 2**). Tumor condition improved significantly with preserved quality of life. Treatment schedule is illustrated in **Figure 4**.

## Discussion

This case highlights the critical balance between efficacy and toxicity management in advanced TSCC. Our patient achieved sustained tumor regression through a two-phase regimen of tislelizumab, lenvatinib, PM-PTX, with or without carboplatin. Notably, the patient reported high satisfaction with treatment outcomes, citing effective tumor control without significant decline in quality of life or physical discomfort throughout the therapeutic course. This outcome underscores the potential of personalized chemo-immunotherapy combinations for aggressive malignancies.

PD-1 blockade reshapes the tumor microenvironment (TME) by disrupting PD-1/PD-L1 interactions. This mechanism enhances anti-tumor immunity through dual pathways: activating effector T cells, B cells, and NK cells while simultaneously depleting immunosuppressive regulatory T cells (Tregs) and myeloid-derived suppressor cells (MDSCs) (9, 10). These coordinated actions increase tumor-infiltrating lymphocytes and improve antigen presentation, ultimately driving robust anti-tumor responses (11).

Our analysis of published case reports/series and clinical trials of immunotherapy in thymic tumors (both first-line and later-line settings) reveals a complex efficacy-safety profile (**Table 1**). While immunotherapy demonstrates clinical activity across treatment lines, thymic malignancies exhibit particularly high rates of severe multi-organ irAEs. Notably, a phase II clinical trial demonstrated that pembrolizumab monotherapy had a 15% incidence of Grade 3–4 irAEs, including immune-related hepatitis, myocarditis, thyroiditis, colitis, conjunctivitis, and nephritis (5). These irAEs increase complexity and cost while compromising survival benefits, quality of life and treatment adherence.

**Table 1:** Summary of published cases and trials of immunotherapy in thymic tumors.

Therapy line	Study type	Tumor type	Regimen	Response	irAEs	Reference
First-line	Case report (n=1)	Advanced thymic carcinoma	Carboplatin + nab-PTX + pembrolizumab	PR (100%, 1)	None	(25)
	Case series (n=2/8)	Advanced thymic carcinoma	Tislelizumab	PR (100%, 2)	None	(26)
Second-line and later	Phase II (n=40)	Recurrent thymic carcinoma	Pembrolizumab	CR (3%, 1) PR (20%, 8) SD (53%, 21)	Severe cardiomyositis (7.5%, 3) Severe hepatitis (10%, 4) Myasthenia gravis (2.5%, 1)	(5)
	Phase II (n=33)	Thymic carcinoma (78.7%, 26) Thymoma (21%, 7)	Pembrolizumab	PR (19.2%, 5) SD (53.8%, 14)	Hepatitis (12.1%, 4) Myocarditis (9.1%, 3) Myasthenia gravis (6.1%, 2) Thyroiditis (3.0%, 1) Antineutrophil cytoplasmic Antibody-associated rapidly Progressive glomerulonephritis (3.0%, 1) Colitis (3.0%, 1) Subacute myoclonus (3.0%, 1).	(27)
	Phase II (n=53)	Advanced/relapsed type B3 thymoma or thymic carcinoma	Nivolumab ± ipilimumab	PR (14%, 7) SD (53%, 26) PD (27%, 13)	Neutropenia (2%, 1) Immune-mediated transaminitis (2%, 1) Myocarditis (4%, 2)	(28)
	Case report (n=1)	Advanced B2/B3 thymoma	Nivolumab	CR (100%, 1)	Myocarditis (100%, 1)	(29)
	Case report (n=1)	KIT-mutated thymic carcinoma	Pembrolizumab	PD (100%, 1)	None	(30)
	Case report (n=1)	Metastatic thymic carcinoma	Carboplatin + pemetrexed + pembrolizumab	PD (100%, 1)	None	(31)
	Case report (n=1)	Thymic squamous cell carcinoma	Pembrolizumab	SD (100%, 1)	None	(32)
	Case report (n=1)	Metastatic thymic carcinoma	Pembrolizumab	PR (100%, 1)	None	(33)
	Case report (n=2)	Thymic epithelial tumors	Gemcitabine+ carboplatin+ sintilimab (50%,1); or docetaxel+ cisplatin + tislelizumab (50%,1)	SD (50%, 1) PD (50%, 1)	Immune myocarditis (100%, 2)	(34)
	Case series (n=6/8)	Advanced thymic carcinoma	Tislelizumab	SD (50%, 3) PR (50%, 3)	Adrenal insufficiency (12.5%,1) Thyroid dysfunction (12.5%,1) Pneumonia (12.5%,1)	(26)
	Case report (n=1)	Thymic epithelial tumors	Pembrolizumab	PR (100%, 1)	Liver and kidney dysfunction (100%, 1) Hypothyroidism (100%, 1) Myocarditis (100%, 1)	(35)

irAEs, immune-related adverse events; CR, Complete response; PR, partial responses; SD, stable disease; PD, disease progression.

For unresectable or recurrent/metastatic thymic carcinoma beyond first-line therapy, identifying irAE-prone populations is critical. TSCC patients with chronic obstructive pulmonary disease (COPD), autoimmune diseases, or long-term immunosuppressive therapy exhibit heightened susceptibility to irAEs such as pneumonitis (12). Furthermore, combined targeted therapy may further increase irAE incidence despite enhancing anti-tumor immunity. These factors enable pretreatment risk assessment and preventive strategy implementation.

Chemotherapy exerts dual effects on immunity. While primarily inhibiting tumor proliferation via DNA disruption, its non-selective cytotoxicity significantly suppresses immune function by depleting rapidly dividing lymphocytes, particularly effector T cells, B cells, and NK cells. For instance, peripheral T cells of cancer patients can decrease by up to 75% following cyclophosphamide chemotherapy (13). Paradoxically, this systemic immunosuppression may mitigate excessive immune activation induced by immunotherapy, thereby reducing irAE risk. Simultaneously, chemotherapy enhances tumor-specific immunity through immunogenic cell death (ICD). Agents like oxaliplatin release tumor antigens and damage-associated molecular patterns (DAMPs) that activate CD8+ T cells, while selectively eliminating immunosuppressive MDSCs via ROS-induced apoptosis (14, 15). In this case report, the patient with TSCC received three cycles of chemotherapy before initiating immunotherapy, which aids in activating the immune system. Therefore, the balance of systemic suppression and localized activation within tumors coordinates therapeutic effects.

Critically, the combination of chemotherapy with immunotherapy has demonstrated synergistic efficacy, facilitates the formation of immune memory, and is associated with reduced irAEs versus monotherapy (16). KEYNOTE trials demonstrate PD-1 inhibitors combined with chemotherapy reduce irAE incidence versus PD-1 inhibitors monotherapy (17, 18). In our case, chemotherapy’s lymphocyte depletion likely counterbalanced immunotherapy-induced hyperactivation, explaining the absence of severe irAEs despite prolonged therapy.

Notably, Lenvatinib remodels TME through dual mechanisms. It normalizes tumor vasculature by inhibiting VEGFR/FGFR signaling, reducing vascular endothelial-cadherin density and enhancing T-cell infiltration. Concurrently, it depletes immunosuppressive cells (e.g., tumor-associated macrophages and MDSCs) and promotes M1 macrophage polarization (19, 20). Critically, Lenvatinib downregulates exhaustion markers (PD-1, TIM-3) on cytotoxic T lymphocytes by restoring IFNγ-mediated JAK/STAT signaling through FGFR suppression (21). These immunomodulatory effects not only augment anti-PD-1 efficacy but also mitigate irAEs by tempering macrophage-derived excessive inflammation.

Lenvatinib’s role in this regimen is supported by the single-arm phase 2 REMORA trial and the recent long-term follow up, which reported a 38% ORR (90% CI 25.6-52.0) and 28.3-month median OS (95% CI 17.1-34.0) in platinum-pretreated unresectable advanced or metastatic thymic carcinoma patients receiving lenvatinib monotherapy (24 mg/day), though with 64% incidence of grade ≥3 hypertension (6, 7). Our modified dosing (8 mg/day) with close monitoring likely contributed to maintained efficacy while avoiding significant hypertension. This contrast underscores the advantage of combination regimens in optimizing safety while maintaining efficacy.

Additionally, our triple combination finds support in translational and clinical precedents. The PECATI trial demonstrated that lenvatinib plus pembrolizumab shows promising activity in pretreated thymic tumors, while studies in hepatocellular carcinoma have established the efficacy and safety of tislelizumab plus lenvatinib combination therapy (22, 23). These findings provide translational rationale for our triple combination regimen.

The strategy of balancing chemo-immunotherapy involves several key aspects. Firstly, baseline differences such as nutritional status and mental state can significantly influence their response and tolerance of treatment. Therefore, personalized strategy should be formulated based on various factors, including the patient’s PS score, pathological type, TNM stage, and PD-L1 expression level. Secondly, the sequence of administration in combination therapy is crucial. Administering chemotherapy drugs prior to immunotherapy may yield favorable therapeutic effects by prematurely activating the immune system. Thirdly, the selection of chemotherapy and immunotherapy drugs is critical, as opting for multi-target chemotherapy agents can enhance the efficacy of immunotherapy. Fourthly, the incidence and severity of adverse reactions may differ among various immunotherapy agents, making it essential to promptly transition to those that provide improved safety and tolerability for patients. In suitable cases, de-escalation of immunotherapy may be considered, such as shifting from a dual-target CTLA-4 + PD-1 bispecific antibody to a PD-1 monoclonal antibody (24). Fifthly, close monitoring and management during the treatment process may help promptly identify and address potential irAEs. Finally, it is important to emphasize the timely and individualized adjustment of chemotherapy drug dosages. Low doses of specific chemotherapeutic agents can modify TME thereby enhancing the efficacy of immunotherapy while minimizing toxicity.

In this case, during the second phase of immunotherapy, carboplatin was omitted, and only PM-PTX were administered. This strategy not only decreased the cumulative toxicity associated with carboplatin but also maintained a balance between chemotherapy and immunotherapy, thereby sustaining the antitumor effect and facilitating a continuous reduction of tumor burden.

## Conclusion

This case supports tislelizumab + lenvatinib + PM-PTX as an effective regimen for advanced TSCC after platinum stabilization, providing tumor control with preserved quality of life. Consider this approach when first-line response is suboptimal, with vigilant toxicity monitoring and regimen tailoring to maintain safety.

## Data Availability

The original contributions presented in the study are included in the article/**Supplementary Material**. Further inquiries can be directed to the corresponding authors.
