# Supplementary material for: Case report and literature review: tislelizumab combined with lenvatinib and polymeric micellar paclitaxel for thymic squamous cell carcinoma
Source: Front Immunol. 2025 Sep 18;16:1677723. doi: 10.3389/fimmu.2025.1677723 (PMC12488663; doi:10.3389/fimmu.2025.1677723)
Supplement: Supplementary file 1 [file Table1.docx]

**Supplementary Table 1：**Biomarker and Next-Generation Sequencing Results​

| **Parameter** | **Method** | **Result** |
| --- | --- | --- |
| PD-L1 expression | IHC | TPS<1%, CPS<1 |
| Microsatellite Status​ | NGS | Microsatellite stable (MSS) |
| Tumor Mutational Burden​ | NGS | Not detected |
| Gene Alterations​ | NGS | No clinically significant variants detected |

Note: TPS, tumor proportion score; CPS, combined positive score; NGS, Next-generation sequencing.

**Supplementary Table 2:**  Supplementary Table 2. Summary of Adverse Events and Management​

| **Adverse Event** | **CTCAE Grade** | **Clinical Manifestations** | **Intervention** |
| --- | --- | --- | --- |
| Skin rash | 1 | Localized rash on forearms | No required |
| Abdominal pain | 2 | Mild-moderate abdominal discomfort | Progesterone treatment |
| Immune hepatitis | 2 | Mildly Elevated transaminases | Hepatoprotective agents |

Note: All adverse events were managed without treatment discontinuation. No Grade ≥3 events occurred.
